# The Management of Type 2 Diabetic Patients with Hypoglycaemic Agents

**DOI:** 10.5402/2012/478120

**Published:** 2012-05-07

**Authors:** Man-Wo Tsang

**Affiliations:** Diabetes Ambulatory Care Centre, Department of Medicine and Geriatrics, United Christian Hospital, 130 Hip Wo Street, Kwun Tong, Hong Kong

## Abstract

Type 2 Diabetes Mellitus (T2DM) is characterized by chronic hyperglycemia with disturbance in carbohydrate, lipid, and protein metabolism due to insulin resistance and beta cell dysfunction. Epidemiological studies have confirmed a global pandemic of T2DM, which has created an enormous burden on society, with regard to morbidity, mortality, and health care expenditures. Life style modifications are fundamental not only in early stages of disease management but need to be intensified as disease progresses. United Kingdom Prospective Diabetes Study (UKPDS) has demonstrated the progressive nature of T2DM, and as disease progresses, a combination agents—oral antidiabetic drugs (OAD) and insulin—are needed in order to maintain good sugar control. The general consensus of HbA1c target for most patients is less than 7%, and various guidelines and algorithms have provided guidance in patient management to keep patient at goal. As our understanding of pathophysiological defects advances, targeting treatment at underlying defects not only enables us to achieve HbA1c goal but also reduces morbidities, mortalities, and progression of the disease. Traditional oral agents like metformin and sulfonylureas have failed to arrest the progression of T2DM. New agents such as TZD, DPP-4 inhibitor, and SGLT-2 may increase our armamentariums against T2DM.

## 1. Pathophysiology of T2DM

Both genetic and environmental factors play an important role in the pathogenesis of T2DM. The best studied pathophysiological defects in T2DM are insulin resistance and insulin secretary dysfunction of *β*-cell [1]. The former is primarily represented by decreased insulin-stimulated glucose uptake in skeletal muscle, unsuppressed hepatic glucose production, and increased lipolytic activity in adipose tissue. The latter is an apparent progressive process with both functional defects in islet cell function and, eventually, apparent loss of *β*-cell mass [2]. In Pima, Indian insulin resistance proceeds beta cell dysfunction while in others beta-cell dysfunction starts early in the natural history of disease progression [3]. The difference probably related to genetic factors. Recent studies also found that a dysfunction of glucagon secretion and impaired incretin system contribute to hyperglycemia in T2DM, which will provide untapped potential for the betterment of diabetes care [4–6]. 

## 2. Current Oral Antidiabetic Drugs (OADs)

Currently six classes of OADs are available, and a new one is around the corner. They can be classified into insulin secretagogue (sulfonylureas, meglitinides), insulin sensitizer (thiazolidinediones), decrease glucose flux (alpha-glucosidase inhibitors), incretin mimetic agent (DPP-4 inhibitor), and glycosuric agent (Table 1). An important message from the table is that not a single agent is effective in tackling all the pathophysiological defects of T2DM.

### 2.1. Sulfonylurea (SU)

SUs have played an important role in hyperglycemia management because of their potency, fast action, and relative low cost. The United Kingdom Prospective Diabetes Study (UKPDS) results confirmed that first-line therapy with sulfonylureas in newly diagnosed T2DM is a safe and effective treatment for glucose control [7]. SUs work by stimulating insulin secretion; although there is evidence of extra pancreatic effect, the clinical effect is probably insignificant [8]. The first-generation agents (acetohexamide, chlorpropamide, tolazamide, and tolbutamide) have a lower binding affinity to the receptor on the *β*-cells, so they must be given in higher doses than the second generation agents, (glimepiride, glipizide, gliclazide, and glyburide) which have a higher binding affinity. Among the second generation agents, there are difference in their differential binding specificity to beta-cell SUR1 and SUR-2 in cardiac muscle [9]. All the SUs act by binding to the SUR-1 subunit of KATP channels, causing them to close and increase intracellular potassium, which triggers membrane depolarization. Membrane depolarization opens up calcium channel and causes influx of calcium. Increase in intracellular calcium stimulates migration and exocytosis of insulin granules [10]. Differences in insulin secretory characteristics of the various insulin secretagogues depend on their pharmacokinetic and the affinity and kinetics of their binding to SUR-1 subunit. They have comparable efficacy as illustrated in Table 2. The common side effects are hypoglycemia, weight gain, and secondary failure [11]. Secondarily SU failure rate is reported to be around 5 to 10 percent of patients per year [12]. Secondary failure can have many causes including progression of the disease, stress, infection, introduction of other drugs, for example, corticosteroids, noncompliance, or nonadherence to diet and exercise. Most of the hypoglycemic effects of the sulfonylureas will be observed at one half of the maximum dose recommended for a specific agent [13]. 

Ever since tolbutamide was implicated with increased mortality secondary to cardiovascular events in the University Group Diabetes Program (UGDP) study, debate on SU cardiovascular safety continues [14]. Recent population studies [15, 16] reported increased coronary heart event and mortality with increased dose of SU exposure, and plausible mechanisms have been related to blockage of SUR-2 receptor in myocardium and impaired the preischemic precondition of myocardium [17]. While the cardiovascular adverse effects associated with SUs remain controversial, it would be rational to dose SUs at the lowest therapeutically effective dose, thus avoiding the loss of selectivity of these agents for pancreatic KATP channels. Sulfonylureas should be started at low doses and titrated up every 1 to 4 week. A linear dose-response relationship does not exist throughout the manufacturers' dose range for SUs [18]. In patients who are not responding at one half of the maximum dose, an alternative agent or combination therapy should be considered. Combining a drug that increases insulin secretion with one that improves insulin action is therapeutically worthwhile.

### 2.2. Nonsulfonylurea Insulin Secretagogue

#### 2.2.1. Repaglinide/Nateglinide

Nonsulfonylurea insulin secretagogue has a mechanism of action that is similar to SU. They bind to kir-6.2 subunit of SU receptor of *β*-cell [10]. Characteristics of these group of agents include a rapid action and short duration of action. The ability to titrate time and dose of the medication to match meal ingestion time greatly decreases in postprandial sugar surge and decreases risk of hypoglycemia. They are good for patients with an irregular meal pattern as they allow greater flexibility for the patient in terms of meal time and dose adjustment. It is to be taken within 30 minutes of each meal with an extra tablet for extra meal and skip a tablet if a meal is skipped. Nonsulfonylurea insulin secretagogues are metabolized by the liver, and although there are no contraindications for patient with renal impairment, the dose should be reduced in cases of impaired liver disease. In general, efficacy is comparable to other SUs with repaganides but less with nateglinides (Tables 2 and 3). Side effects are similar to SUs but less weight gain. Hypoglycemia is uncommon and is usually mild. In the most recent diabetes prevention trial, “*The nataglinides and valsartan in impaired glucose tolerance outcome research (navigator) study*”, Nateglinide have not been proved to have a benefit on any cardiovascular outcome [19]. 

### 2.3. Thiazolidinediones

Pioglitazone belongs to the class of thiazolidinediones and is an activator of the nuclear transcription factor, peroxisome proliferator-activated receptor-*γ* (PPAR-*γ*), which modulates the activity of a host of genes that regulate carbohydrate and lipid metabolism. Its major actions are to increase insulin-mediated glucose uptake (improves insulin sensitivity) in muscles, increases adipogenesis, preserves beta cell function, and modulates hepatic gluconeogenesis. The first-generation thiazolidinediones, troglitazone, were withdrawn from the market because of hepatotoxicity and the second-generation, rosiglitazone, is the only in restricted market because of suspected cardiac side-effect. Pioglitazone is the only drug of the class still widely available. It has moderate efficacy in lowering fasting blood sugar and HbA1c [20]. It has a favorable effect on lipid profile, decreases plasma triglyceride, and increases high-density lipoprotein [10]. In PRO-ACTIVE study, pioglitazone has shown to reduce composite of all-cause mortality, nonfatal myocardial infarction, and stroke in people with T2DM who have a high risk of macrovascular events [21]. Studies with thiazolidinediones in prediabetes, impaired glucose tolerance (IGT), impaired fasting glucose (IFG), had demonstrated significant reduction in progression from IGT to T2DM by 62–72% [22–24]. It is more effective in obese subjects and should be used early in the treatment of patients with T2DM to delay disease progression and to minimize the development of complications [25]. 

Hypoglycemia caused by pioglitazone is usually mild unless in combination with SU and most common side-effect is fluid retention. Though the incidence of congestive heart failure in pioglitazone treated patients is very low, the risk increases from 1% to 4-5% in patients already treated with high dose insulin and pioglitazone [26]. A patient with advance heart failure, New York Heart Association stage III/IV, is a contraindication for pioglitazone. Retrospective analysis of rosiglitazone and pioglitazone data pool revealed that diabetes using thiazolidinediones had a higher risk of distal upper and lower limb fractures compared with those not using thiazolidinediones. Fracture proportions were higher among women and increased with age. The observed excess risk of fractures for women in the pharmaceutical company data set on pioglitazone is 0.8 fractures per 100 patient-years of use [27]. The risk of bladder cancer from recent epidemiological data had prompted France and Germany to suspend pioglitazone in early 2011. In July 2011, the European Medicines Agency's Committee for Medicinal Products for Human Use (CHMP) confirmed that Pioglitazone remains a valid treatment option for certain patients with type 2 diabetes but acknowledges that there is a small increased risk of bladder cancer in patients taking these medicines and warns not to use these medicines in patients with current or a history of bladder cancer or in patients with uninvestigated macroscopic haematuria [28].

### 2.4. Biguanide (Metformin)

The major target of metformin is the enzyme AMP-activated protein kinase (AMP-kinase). Activation of AMP-kinase by metformin results in decrease of hepatic glucose production and increase glucose transport in skeletal muscle [29]. The overall effect is a decrease in hepatic gluconeogenesis due to improvement in hepatic insulin sensitivity. Its insulin sensitizing effect on peripheral tissue has been minimal only. Its efficacy in glucose control had been well documented in UKPDS. Metformin use in the newly diagnosed T2DM achieved comparable HbA1c lowering to SU but without weight gain. Use in obese subgroup in UKPDS was associated with improvement in cardiovascular outcome. The risk of myocardial infarction was reduced by 39% and the overall diabetes-related mortality by 42% [30].

Most frequent side-effects are related to the gastrointestinal, tract, namely, nausea, poor appetite, abdominal discomfort, and diarrhoea. Long-term use has also been associated with vitamin B12 deficiency [31, 32]. While the most feared lactic acidosis (LA) is actually quite rare and mostly occurred in clinical situations where metformin use is contraindicated, the reported incidence of lactate acidosis in patients with metformin is 3 per 100,000 patient years and a recent Cochrane review suggested there is no evidence that metformin is associated with an increased risk for lactic acidosis when prescribed under the study conditions [33]. The great majority of cases of metformin-associated LA occur in connection with acute illness in diabetic patients where cardiac, hepatic, pulmonary, or renal function is compromised. There are always at least two predisposing factors present in these instances. It is, therefore, reasonable to assume that metformin is just a “bystander” [34].

Metformin is recommended by many algorithm/guidelines as the first-line treatment and can be combined with other oral hypoglycaemic agents with complementary action. In order to increase patient's tolerance, it should be started with a low dose and increase gradually over weeks. Maximum dose is 2500 mg per day [35]. It is claimed that sustained preparation is associated with better patient compliance and better HbA1c improvement [36]. The drug should be used with caution in elderly and patients with liver or renal impairment. It is contra-indicated in chronic alcoholism and creatine clearance less than 50 mL/min. It should be stopped for two days before contrast studies [36].

### 2.5. *α*-Glucosidase Inhibitor (Miglitol and Acarbose)

The mechanisms of all the *α*-glucosidase inhibitors are similar, as a competitive inhibitor to the oligosaccharides for the binding site of *α*-glucosidase. They must be given at the start of each meal. They must be started with a low dose and titrate gradually within weeks. They mainly reduce postprandial hyperglycaemia. The mean reduction in diet control T2DM is about 3.0 mmole/L and HbA1c 0.9% [37]. The most frequent side-effects of acarbose treatment are flatulence and diarrhoea. They can be used with T1DM orT2DM. In STOP-NIDDM trial, acarbose not only prevented new diabetes mellitus development but also suggested a reduction in hypertension and cardiovascular disease [38].

### 2.6. Dipeptidyl Peptidase 4 (DPP-4) Inhibitors

People with T2DM are known to have deficient meal-related incretin responses [4, 5], resulting in decreased insulin secretion, increased postprandial glucagon levels, and elevated postprandial glucose [39]. This has led to the development of a new class of drug call incretinmimetics, which are GLP-1 analogue or GLP-1 receptor agonist and DPP4 inhibitors. The former can only be given by injection while the latter are orally active [40].

The highly selective DPP-4 inhibitors, sitagliptin, saxagliptin, vildagliptin, and linagliptin, prevent normal rapid degradation of endogenous glucagon-like-peptide-1 (GLP-1). They are selective because they inhibit DPP-4 significantly more than the related enzymes, DPP-8, and DPP-9. GLP-1 and glucose-dependent insulinotropic polypeptide (GIP) half-lives and protein levels are dramatically increased when DPP-4 inhibitors are administered. These drugs reduce postprandial and fasting glucose concentrations with sustained decrease in HbA1c (0.7–1.3%) without weight gain or significant hypoglycemia [41]. They potentially preserve *β* cell function with chronic use and have favorable safety profiles. Neither weight loss nor nausea occurs with DPP-4 inhibitors. The most commonly reported adverse events have been mild infections such as nasopharyngitis, upper respiratory tract infection, and headaches. No clinically relevant changes in laboratory immunologic parameters have been found in studies of DPP-4 inhibitors, and pancreatitis was reported at lower rates with the DPP-4 inhibitors compared with other oral antidiabetic agents [42].

### 2.7. SGLT-2 Inhibitor: Dapagliflozin

A new approach in management of hyperglycemia, as inspired by the congenital familial renal glycosuria [43], is by inhibiting renal glucose reabsorption. SGLT-2 is specific glucose transporter in the proximal renal tubules. SGLT-2 inhibitors, such as dapagliflozin, have been in clinical trials to prove clinical application of these agents [44]. Use of SGLT-2 inhibitor results in glycosuria in the order of 30–80 gm/day, eliminating glucose from the circulation and the equivalent energy. Recent data suggest that it has a moderate HbA1c lowering effect 0.5–0.8% [45]. Dapagliflozin has demonstrated efficacy, alone or in combination with metformin, in reducing hyperglycemia in people withT2DM [44, 46].

It is metabolized by the liver and can be used in patients with renal problem. Their mechanism of action is independent of beta cell or insulin resistance. They can be added to other oral antidiabetic drugs. Potential problems with SGLT-2 inhibitor are risk of urinary tract infection and diuretic effect of glycosuria. Additional clinical studies are needed to prove their safety and long-term effect in natural progression of T2DM and cardiovascular complication development [47].

## 3. Treatment Target and Guidelines/Algorithm

In general, HbA1c < 7% is the commonly accepted target, but in selected population, HbA1c < 6% is suggested [48, 49]. A lower or near normal HbA1c may be a good target for younger patients with a shorter duration of T2DM and those with no history of cardiovascular disease when one hopes to prevent coronary heart disease [50].

Different associations, ADA/EASD, AACE/ACE, NICE, have published different guidelines in diabetes management [51–53]. Most of these adopt a stepwise approach with life style modifications, exercise, and medical nutrition therapy, as the first step, followed by metformin and other oral hypoglycemic agents or insulin in subsequent steps. They differ in the second-line agents recommended, and this has caused confusion among practitioners with different cultural, societal, and economic development.

Instead of a conventional stepwise approach, the DeFronzo algorithm recommends metformin, pioglitazone, and exenatide (GLP-1 agonist) as initial comprehensive treatment [54]. The triple therapy will work complementary to each other with the advantage of low risk of hypoglycemia, no weight gain, and potential coronary heart disease risk protection, and prevention of beta cell function deterioration. Definite proof of the therapy will come after completion of the study, which is funded by ADA recently.

## 4. Strategy

**Figure 1:**
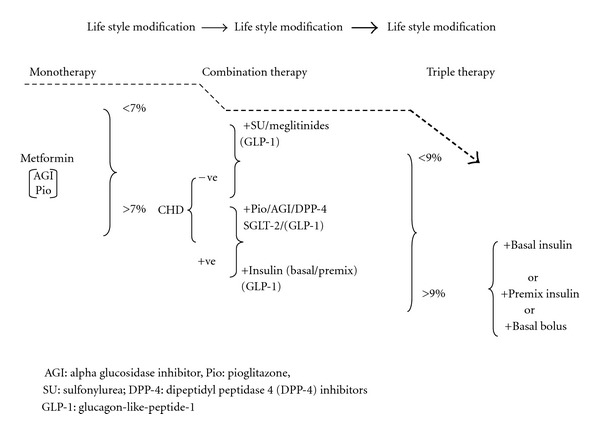
Algorithm in management of T2DM. NICE guidelines, May issue 2009 [53], Nathan et al. [51], Rodbard et al. [49], and Inzucchi and McGuire [65].

**Figure 2:**
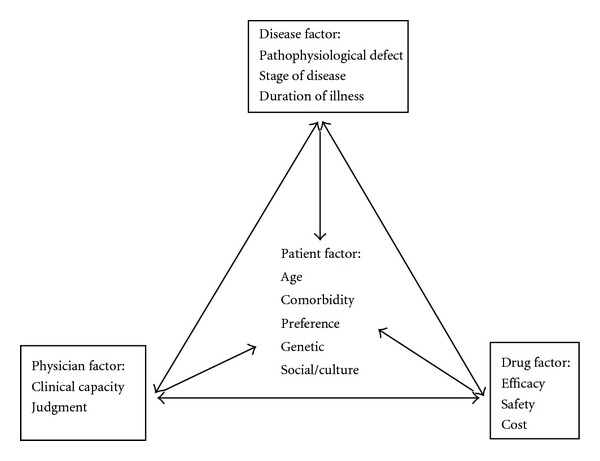
Interaction between different factors in choosing an appropriate agent for T2DM management.

A uniform treatment protocol is impossible for all regions and no one protocol fits all patients. After life style modifications, pharmaceutical treatment usually starts with monotherapy, unless the patient is very symptomatic. If adequate blood glucose control is not attained using a single oral agent after 3–6 months, a combination of agents with different mechanisms of action may have additive therapeutic effects and result in better glucose control. Further deterioration is to be expected with time, and insulin in various combinations will be required ultimately if tight control of blood sugar is required. Potential combinations are illustrated in Figure 1. In practice, management of people with T2DM will depend on consideration of at least four different factors; patient, disease, drug, and physician as depicted in Figure 2.

### 4.1. Disease

Treatment strategy is to address the pathophysiological defects and aims at correcting one or more of these physiologic abnormalities, that is, insulin resistance, beta-cell dysfunction, and increased hepatic glucose output, and not simply on the reduction in HbA1c. This will imply use of different drugs or combination of drugs at different stages of the disease. Treatment must be started early in the natural history of T2DM if *β*-cell failure is to be prevented because of the “Metabolic legacy” as demonstrated by UKPDS [1, 55].

### 4.2. Drug: Potency and Safety the New and Old Agents

Metformin and SU have served us well over half century and they are still recommended by various algorithms. However, they failed to sustain glucose control as a result of *β*-cell failure as demonstrated by UKPDS [56]. Hypoglycemia is a major and potential lethal side effect with SUs, especially in elderly and patient with cardiovascular disease (CHD). This can be minimized by dosing at less than the manufacturers' maximal recommended dose and avoiding high risk patients and agent [14–18]. New agents such as pioglitazone and DPP-4 inhibitors may offer less hypoglycemia, potential *β*-cell protection, sustain glycaemic control, and possibly CHD protection in high risk patients. But pioglitazone is associated with significant distal fracture, heart failure, and potential risk of bladder cancer. Though initial clinical data are promising, there are still no long-term safety data about incretin-based treatment.

### 4.3. Patient

Different patients may need different regimens. Genetic and cultural background difference may affect their response and adherence to specific drugs. The comorbid states, such as coronary heart disease and kidney disease, may pose them at particular risk such as heart failure, lactate acidosis, hypoglycemia, and even fatal myocardial events. As we learnt from ACCORD and ADVANCE studies that patients with long duration disease or established coronary disease should not have aggressive lowering of blood sugar [57, 58].

### 4.4. Physician

Despite management guidelines recommending increasingly tight targets for glycaemia control, a significant proportion of patients with type 2 diabetes do not achieve target levels of glycaemia control. A number of studies have shown that when targets are lower, a smaller proportion of patients reach target; in China (CODIC-2), 68% had HbA1c < 7.5% [59], Canada, 51% had HbA1c ≤ 7% [60], and USA, only 37% HbA1c had <7% [61]. Europe had 31% HbA1c < 6.5% [62]. In a cross-sectional survey of 24 317 patients with diabetes mellitus among five different Asia countries, the majority (55%) had values exceeding 8%, indicative of poor glycogenic control [63]. There appeared still a gap between what is known and what is being done. Study also found that because of clinical inertia, patients accumulate several years of hyperglycemia before therapy is intensified or changed. Encourage to change to an alternative agent or early combination therapy when most of the hypoglycemic effects are not observed at one half the maximum dose of the sulfonylureas should be the first step to reduce time of exposure to chronic hyperglycemia and possible complication [13, 64]. The decision to use specific agent depends on judgment of physician after balancing all the above factors.

## 5. Conclusion

Conventionally, drug interventions for T2DM have focused on improvements of HbA1c, which proved to be important in prevention of microvascular complication and cardiovascular benefit in long term. However, their efficacy tends to fail as disease progresses. New agents targeting at insulin resistance and *β*-cell protection offer effective regimens to slow disease progression and complication development. Algorithm and guidelines may offer suggestions in choosing appropriate agents for general patient only. Each patient differs with his particulars and how to choose the appropriate agent depends on each practitioner's clinical judgment after taking into consideration the risks and benefits of each agent and unique clinical features of each patient and stages of the disease. Data are gathering to enable us to consider agent or combination of agents to help arrest progression of T2DM and prevent complication.

## Figures and Tables

**Table 1:** Different classes of oral antidiabetic agents (OAD) and clinical indications.

	*α* Glucosidase Inhibitors	Meglitinides	SUs	TZDs	Metformin	DPP-4 Inhibitors	SGLT-2
Insulin deficiency		*✓*	*✓*			*✓*	
Insulin resistance				*✓*	*✓*		
Excess hepatic glucose output				*✓*	*✓*	*✓*	
Intestinal glucose absorption	*✓*				*✓*		
Glycosuria							*✓*

Amori et al. [41];

Abdul-Ghani et al. [47];

DeFronzo [17].

**Table 2:** Mechanism, site of action, and efficacy for oral Antidiabetic agents and CHD benefit.

Drug class	Mechanism of action	Primary site of action	Reduction in FBS	Reduction in HbA1c	CHD benefit
Sulfonylureas*∴*	Insulin release	Pancreas	3.34–3.88	1.0–2.0	−
Nonsulfonylurea secretagogues	Insulin release	Pancreas	3.34–3.88	0.07–2.0	−
Biguanides	Hepatic glucose production; insulin sensitivity in hepatic and peripheral tissues	Liver; peripheral tissues	3.34–3.88	1.0–2.0	+
Thiazolidinediones	Insulin sensitivity in peripheral tissues; hepatic glucose production	Peripheral tissues; liver	1.90–2.22	0.7–1.0	+
Alpha-glucosidase inhibitors	Delay carbohydrate absorption	Small intestines	1.38–1.66	0.5–1.0	+
DDPIV inhibitors*	Enhance endogenous GLP-1	*β*-cell, stomach, liver	0.5–1.0	0.73–1.2	+/−
SGLT-2^#^	Inhibitor of renal proximal tubular reabsorption	Renal tubular SGLT-2 receptor	0.6–1.2	0.37–0.72	+/−

DeFronzo [17]; Nathan [66].

^#^Bailey et al. [45]. *Amori et al. [41].

**Table 3:** Oral Antidiabetic Drug dosage and side effect at a glance.

Drug class	Dose (mg/day)	Advantage	Combination	Side effect	Contraindication
*Sulfonylurea*		Rapid FPG reduction Early stage disease	Biguanide/TZD AGI/DPP-4/insulin	Weight gain, +hypoglycemia	Allergy, DKA Pregnancy, lactation
Glibenclamide	5–20	Low cost			In elderly, liver/renal
Glipizide	10–40				
Gliclazide	80–320				
Glimepiride	1–8	QD			

*Meglitinides*		Fast, flexible dosing	Biguanide/TZD/insulin	Weight gain/hypoglycemia	Allergy, DKA Pregnancy, lactation
Repaglinide	1.5–12	mild weight gain and hypoglycaemia	Biguanide/TZD/insulin		
Nateglinides	60–180	mild weight gain and hypoglycaemia	Biguanide/TZD/insulin		

Biguanide Metformin	1500–2500	No weight gain, CHD benefit −hypoglycemia, FBS	SU/TZD/DPP-4 AGI/insulin	GI upset, vitamin B12 deficiency	LA/renal/hepatic disease; CHF

Pioglitazone (TZD)	15–45	Insulin sparing. Low hypoglycemia CHD benefit	Biguanide/SU/insulin/DPP-4	Edema/weight gain; High cost, Slow onset of action	Heart failure; DKA; liver disease Ca bladder, fracture

*α* Glucosidase inhibitor (AGI) (Acarbose and Miglitol)	150–300	no hypoglycemic FBS/PPG	Biguanide/su/TZD/DPP-4/insulin	High cost GI side effects	GI Cirrhosis; DKA; IBD, CKD

*DPP-4*		Weight, −hypo, +*β* cell	Biguanide/SU/TZD/insulin	URI	Pancreatitis dose adjust in CKD
Sitagliptin	25–100	QD		URI	
Vildagliptin	50–100	BD		URI	Liver disease
Saxagliptin	5	QD		URI	
Linagliptin	5	QD, no dose adjust CKD		URI, stuffy nose	

*SGLT-2*: Dapagliflozin		Early and late stage of disease efficacy independent of beta cell and IR	All OADs	Urinary tract infection	

OAD: oral antidiabetic agent, DKA: diabetic keto-acidosis, IBD: inflammatory bowel disease, CKD: chronic kidney disease, URI: upper respiratory tract infection, FBS: fasting blood glucose, PPG: postprandial glucose.
